# Implementation of point of care HIV viral load monitoring for people living with HIV in low- and middle-income countries: A systematic review on implementation research outcomes

**DOI:** 10.1371/journal.pone.0313802

**Published:** 2026-02-04

**Authors:** Perry Msoka, Iraseni Swai, Kennedy Ngowi, Ria Reis, Andreja Lekic, Blandina T. Mmbaga, Anita Hardon, Marion Sumari-de Boer

**Affiliations:** 1 Kilimanjaro Clinical Research Institute, Moshi, Tanzania; 2 Amsterdam Institute for Social Science Research, University of Amsterdam, Amsterdam, the Netherlands; 3 Amsterdam Institute for Global Health and Development, Amsterdam, the Netherlands; 4 UMC Amsterdam, location AMC, Amsterdam, the Netherlands; 5 The Children’s Institute, University of Cape Town, Cape Town, South Africa; 6 School of Medicine- KCMC University, Moshi, Tanzania; 7 Kilimanjaro Christian Medical Centre, Moshi, Tanzania; 8 Knowledge, Technology and Innovation Chair group, Social Sciences Department, Wageningen University & Research, Wageningen, the Netherlands; Zambia Ministry of Health, ZAMBIA

## Abstract

**Background:**

Viral load monitoring has rapidly increased among people living with HIV(PLHIV) in low- and middle-income countries (LMICs), resulting in an increased laboratory workload. The use of innovative Point of Care (PoC) or near Point of Care (n)PoC HIV Viral Load (HIV VL) monitoring has enabled improved patient care, a reduction in laboratory workload, improved clinic retention and reduced turnaround time of results. However, implementation bottlenecks of such methods are uncertain, especially when PoC or (n)PoC is implemented in remote areas in low-volume clinics.

**Objectives:**

The main aim of this study was to review implementation research outcomes of (n)PoC HIV VL monitoring for PLHIV in LMICs.

**Methods:**

We qualitatively synthesised peer-reviewed papers to explore implementation research outcomes (IROs) of (n)PoC HIV VL monitoring. We identified studies published between January 2013 and June 2024. We used the IROs described by Proctor et al., which are acceptability, adoption, appropriateness, cost, feasibility, fidelity, penetration and sustainability. We searched using the following Mesh terms: Point of care testing, HIV, viral load, acceptability, patient acceptance of health care, adoption, facilities and services utilisation, appropriateness, cost, feasibility, fidelity, penetration, coverage, sustainability and continuity of patient care through PubMed, Cochrane and Scopus. The PRISMA diagram in the Fig 1 presents the selection process of included papers.

**Results:**

Twenty studies reported implementation outcomes of PoC or (n)PoC HIV VL monitoring. Near PoC HIV VL monitoring using GeneXpert is considered acceptable to patients and healthcare providers. Point of care HIV VL monitoring using mPIMA was feasible as patients received the results the same day. From a health service provider’s perspective, PoC HIV VL monitoring was acceptable because it influenced patients to accept the illness and adhere to medication. Additionally, there was high testing coverage in routine PoC HIV VL monitoring centres. Fidelity was questionable in some settings due to (n)PoC HIV VL monitoring results not being delivered as intended. Additionally, we found in several studies that the (n)PoC costs are higher than standard of care test, USD 54.93 per test, at low testing volume clinics conducting 20VL tests per month compared to costs of USD 24.25 at high testing volume clinics conducting 100VL tests per month, while centralised testing costs USD 25.65 per test. However, costs are expected to be lower when (n)PoC HIV VL monitoring is scaled up and targeted for those at risk.

**Conclusion:**

Implementation of PoC or (n)PoC testing for HIV viral load monitoring is acceptable and feasible and can reach a vast population. However, higher costs, limited fidelity, lower penetration and limited sustainability may hinder using (n)PoC testing in improving patient care and health outcomes. More knowledge and training should be implemented to overcome these challenges.

**Registration Number:**

PROSPERO 2023 CRD42023394668

## Introduction

Globally, an estimated 39.9 million people were living with Human Immunodeficient Virus (HIV) in 2023, of whom 77% of adults and 57% of children accessed antiretroviral therapy (ART) [1]. The Joint United Nations Programme on HIV and AIDS (UNAIDS) aims to reach 95-95-95 targets by 2030 that 95% of people living with HIV (PLHIV) know their HIV status, 95% of diagnosed PLHIV receive antiretroviral therapy (ART), and 95% of PLHIV receiving ART attain viral suppression below 1,000 copies/ml [2]. To reach these goals, the World Health Organization (WHO) emphasizes that all PLHIV receive ART and routine HIV viral load (VL) testing to monitor treatment response, adherence and virologic suppression [3].

However, monitoring the VL of PLHIV on ART in a centralised manner in Low and Middle-Income Countries (LMICs) is still challenging due to limited infrastructure, shortage of skilled staff, loss of samples, an inefficient system of providing results and patients being lost to follow-up [4]. To overcome those challenges, WHO has advocated for integrating Point of Care HIV Viral load (PoC HIV VL) monitoring in HIV care services, allowing faster diagnosis of treatment failure and better management of patients [5].

Point of Care HIV VL monitoring entails testing at the point where healthcare is provided. The results can be given on the same day. Also, it can usually be performed by not highly skilled lab staff [6]. Near Point of Care HIV VL monitoring is testing in laboratories close to treatment facilities, while still improving the turnaround time from sampling to result compared to centralised testing [7]. The current PoC HIV VL assays that are commercially available or in late development include the PoC HIV assay of mPIMA by Abbott, and the (n)PoC HIV assays SAMBA by Diagnostics for the Real World and GeneXpert HIV 1 by Cepheid [8].

Several studies have been conducted on the performance of PoC HIV VL monitoring. In Mozambique, mPIMA was found to have a high sensitivity of 95% and specificity of 96.5% to identify virological failure at the threshold of 1000 copies/ml when a standard conventional plasma test was used as the golden standard [9]. In addition, a study evaluating the performance of SAMBA in Malawi and Uganda found it to be accurate in differentiating patients who had a VL below or above 1000 copies/ml [10].

Furthermore, a systematic review study from developed and developing countries found that PoC testing is preferable to centralized testing, with a sensitivity of 93.3%− 100% and specificity of 99.5%− 100% for Early Infant Diagnosis (EID), acute HIV infection diagnosis, or VL monitoring [11].

Despite the good performance of PoC HIV VL monitoring in previous studies, several challenges exist. A systematic review of the performance and clinical utility of PoC HIV viral load testing in LMICs observed that there is a need for constant electricity for the machine to function and a temperature control room for sample processing and storage [11].

Other reported challenges of PoC HIV VL monitoring are device operation by not highly skilled lab staff, routine device maintenance, and the high costs of a PoC test [4,6,12]. All these may hamper the implementation of PoC HIV VL monitoring.

Given the above-reported challenges, systematic analysis of studies that explore implementation outcomes and solutions to overcome possible implementation challenges is potential. Proctor and colleagues (2011) designed a framework that describes eight distinct implementation outcomes to guide the successful implementation of innovations: acceptability, adoption, appropriateness, feasibility, fidelity, implementation costs, penetration and sustainability. Acceptability is the perception among stakeholders that a given treatment, service, practice, or innovation is agreeable, palatable, or satisfactory. Adoption is the intention, initial decision, or action to try or employ an innovation or evidence-based practice. Appropriateness is the perceived fit of the innovation to address a particular issue or problem. The cost is the financial effect of an implementation effort. It may include charges for treatment delivery, implementation strategy and use of the service setting. Feasibility is how a new treatment or innovation can be successfully used or carried out within a given agency or setting. Fidelity is defined as the extent to which an intervention was implemented as prescribed in the original protocol or as the program developers intended. Penetration is integrating practice within a service setting and its subsystems. Sustainability is how a newly implemented intervention is maintained or institutionalised within a service setting’s ongoing, stable operations. These implementation research outcomes can give insights into challenges and assist in developing strategies to be investigated in future pragmatic trials [13].

In this systematic review, we use the Proctor framework to explore implementation research outcomes of (n)PoC HIV VL monitoring for PLHIV in LMICs through a qualitative synthesis of peer-reviewed papers. The specific objectives were to review existing evidence on (1) the acceptability, (2) the adoption, (3) the appropriateness, (4) the cost, (5) the feasibility, (6) the fidelity, (7) the penetration and (8) the sustainability of (n)PoC HIV VL monitoring.

## Methodology

Using the reporting items in systematic review guideline [14] we qualitatively synthesised scientific papers to explore the implementation research outcomes (IROs) of PoC HIV VL monitoring for PLHIV. We used the Proctor framework of IROs and pre-defined topics to define the search terms [13].

### Literature search

The initial broad relevant MESH terms were defined based on the terms ‘PoC testing’, ‘HIV’ and ‘Viral load’. We viewed the proper entry terminology for the IROs: Acceptability, Adoption, Appropriateness, Cost, Feasibility, Fidelity, Penetration and Sustainability. After defining these terms, we searched for the terms in June 2024 using a combination of the MESH and entry terms. A detailed search strategy is presented in S1 Table.

Three databases were searched for publications published between January 2013 and June 2024: (1) PubMed, (2) Cochrane and (3) Scopus. After this, we scanned the reference list of each obtained publication found through the above search for additional publications that could fit our search criteria.

### Study selection

Publications were included based on the following criteria:

- Quantitative, including trials, or qualitative studies.
- Original papers.
- Focus on (n)PoC VL monitoring.
- A publication should at least describe or conclude on one of the IROs as described by Proctor.
- Studies examining IROs in any country were considered without restrictions based on income classification.

We used Covidence software https://support.covidence.org/help/how-can-i-cite-covidence to select and organise manuscripts and data extraction. The publications were identified and imported with a librarian (A) into the software for selecting eligible publications. After collecting publications from PubMed, Cochrane and Scopus, the titles and abstracts of identified studies were screened by two reviewers (PM, MS) for relevance. Any disagreements during the screening of titles and abstracts were resolved through discussion. In case of doubt that a publication met the criteria, it was included for full-text assessment to assess the eligibility. The full-text assessment of the quality and risk of bias of all included publications was performed by two reviewers (PM, MS). We used the Newcastle Ottawa scale (NOS) to assess the risk of bias [15]. The tool assessed the bias in three categories which are: selection, comparability, and outcome or exposure. A star system was used: a score of seven to nine stars was considered a low risk of bias, four to six stars were considered an “unclear risk of bias”, and three or fewer stars were considered a “high risk of bias”. The study was considered of low quality if we found it to have a high risk of bias.

All manuscripts included in the full-text assessment were judged based on the quality of the paper using twenty-seven criteria selected from the PRISMA guidelines for reporting systematic reviews [16]. The PRISMA checklist can be found in S2 Table. The twenty selected criteria covered the following items:

- The abstract was comprehensive.
- The objective did clearly state the specific aims of the study.
- The rationale was based on a well-described literature review.
- The methodology clearly described qualitative or quantitative methods, research setting and participants, methods and data collection procedures.
- Ethical issues were assessed by judging informed consent procedures, voluntary participation, no harm, confidentiality and anonymity.
- The analysis and results had to be realistic to achieve the required study objective.

Exclusion criteria were:

- Studies with outcomes that were not relevant to our review.
- Studies describing interventions outside of our area of interest.
- Studies as well as reviews of papers published before 2013 as PoC VL testing was not widely implemented and limited to pilot evaluations in a research setting [10].
- Protocol studies.

### Data extraction and management

One reviewer (PM) extracted the data. The following information was extracted from the included studies:

- Country where the study was performed
- Study design classified as clinical trials, cohort, qualitative or modelling studies
- Study funding sources
- Population description
- Study period, start and end date
- Inclusion and exclusion criteria
- Methods of recruitment of the participants
- Implementation of research outcomes (IROs) investigated

### Ethical approval and informed consent

The review was registered on the PROSPERO registry for systematic reviews. (PROSPERO 2023 CRD42023394668). The review did not involve collecting new data from human subjects. Therefore, ethical approval and informed consent were not required.

## Results

**Fig 1:**
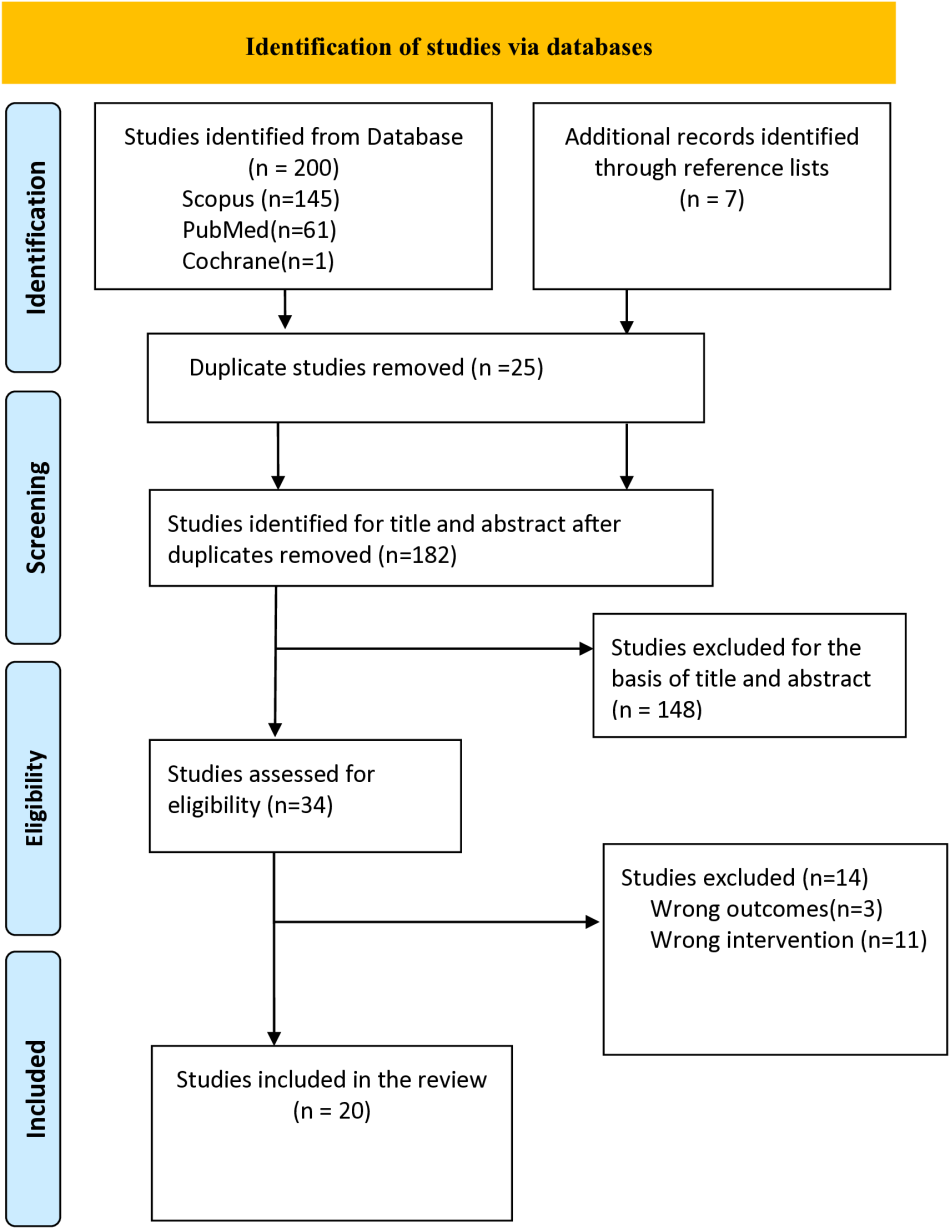
PRISMA diagram of studies screening and selection.

Two hundred studies were found through the initial search of PubMed, Scopus and Cochrane, and an additional seven studies were found through snowball searching of publications of the initial found studies. Twenty-five duplicate studies were removed from the 207 studies. As a result, 182 studies were screened for eligibility based on title and abstract, as demonstrated in S3 Table. From that, 148 irrelevant studies were excluded because the publications focused on something other than (n) PoC VL monitoring. Thirty-four studies were assessed on eligibility by reading the full text of the paper. We found that fourteen studies were not eligible: three had irrelevant outcomes for our review and eleven described interventions outside our area of interest (S4 Table). This led to 20 studies being included in the final review, as shown in Table 1. The PRISMA diagram below shows the selection process (Fig 1).

**Table 1:** Study characteristics.

Author/Year/ Country	Setting	Study Design	N	Duration	Follow-up Period	Data Collection Method	Type of POC Test		Acceptability	Adoption	Appropriateness.	Cost	Feasibility	Fidelity	Penetration	Sustainability	Implementation Issues
							nPoC	PoC									
Boyce et al. (2023) Uganda [28]	Lower-level health centre in rural Uganda	Pilot feasibility study	242 participants	August 2020-July 2021 (12months)	August 2020-July 2021	Quantitative	Gene Xpert		Participants disliked staying at the clinic to receive their results; stigma surrounding VL testing was reflected; there was limited knowledge among both participants and providers regarding its importance	N/A	N/A	N/A	The authors conclude that the implementation of PoC is feasible based on the overall results of the study. They did not see utility disruptions or supply shortfalls	A big part of the ordered tests was not completed because participants did not stay to have blood drawn. Others did not wait for their results.	N/A	N/A	Feasibility was limited due to the presence of invalid results due to power interruptions mid-assay, insufficient sample being added to the cartridge and one was caused by a poorly processed sample (i.e., mixed with red cells)
Reif et al.,(2022) Haiti [36]	HIV/AIDS Clinic	Randomized control trial	150 participants	May 2018-April 2019	6 months	Quantitative	GeneXpert		N/A	N/A	N/A	N/A	According to the authors, POC was efficiently integrated in care	N/A	N/A	N/A	Delays in same-day POC VL results, clinic closed before the assay process was completed; participants’ late arrival at the clinic; stockouts of cartridges
Boeke et al., (2021) Cameroon, Congo, Senegal, Kenya, Tanzania, Zimbabwe, Malawi [27]	57 facilities across 7 countries in Sub Saharan Africa	Retrospective evaluation of routine data	6795 nPOC vs 17, 614 SOC	2017-2019(country dependent)	90 days	Quantitative	GeneXpert	mPIMA	N/A	N/A	N/A	N/A	N/A	Low fidelity was reported due to several system gaps, including poor result documentation, inadequate patient communication and follow up and limited clinical capacity to switch patients to second-line therapy	N/A	N/A	N/A
Bulterys et al (2021) Kenya [17]	5 Ministry of Health facilities	Micro costing (Opt4kids RCT, Opt4Mamas cohort)	Opt4Kids randomized; Opt4Mamas 700	March 2019-Jun 2020	Opt4kids 12months; Opt4Mamas 6months	Mixed method	GeneXpert		N/A	N/A	N/A	High (n)PoC test costs USD 54.93 in low-volume clinics and a low-cost USD 24.25 in high-volume clinics, with centralised testing cost of USD 25.65 per test	The authors conclude that PoC can be feasibly implemented. But there is low feasibility in some settings that shared the same instrument causing delays in VL results	N/A	N/A	N/A	N/A
Guenguen et al (2021) Malawi, Uganda. [31]	5 HIV Clinics +Arua RRH	Descriptive analysis	60,889 samples	Aug2013-Dec 2016	Not defined	Quantitative	SAMBA 1 VL		High satisfaction of (n)PoC test as it minimized the frequency of clinic visits for high-risk patients	N/A	N/A	Implementing (n)PoC testing in numerous remote health facilities is perceived as unrealistic and too costly for Ministries of Health	(n)PoC testing was practical and effective despite logistical and staffing challenges; the authors concluded that SAMBA I VL is feasible in peripheral facilities and district hospitals	It was found that only 2% of test results for patients seen at one site were reviewed with the patient on the same day. It took more than one year to adapt to the new monitoring paradigm based on VL due to insufficient training	Low penetration of (n) PoC due to manpower insufficient and high workload. But it is mentioned that task shifting to community health workers may overcome these challenges	Low sustainability of (n)PoC test in clinics with challenges of staff availability and unreliable power supply	N/A
Sharma et al (2021) South Africa. [19]	Durban public clinic	Modelling study (STREAM RCT)	390 RCT; modelled 175, 000+	Feb 2017-Oct 2018	12 months	Quantitative	GeneXpert		N/A	N/A	N/A	The intervention is cost-effective in moderately sized clinics	PoC testing combined with client centered care, including referral of stable clients to DSD, can efficiently improve patient outcomes and reach UNAIDS ambitious treatment targets in SSA	N/A	N/A	N/A	N/A
Wang et al. (2021) Malawi and Zimbabwe. [23]	Public sector health facilities (10 in Malawi, 8 in Zimbabwe)	Pre and post implementation study	10, 272 tests across all facilities and test types	Nov 2016-Feb 2018	No long-term follow-up; outcomes assessed within the study period	Quantitative	GeneXpert		N/A	N/A	N/A	N/A	Multi disease testing was highly feasible to scale up HIV testing. But site selection should be based on data to ensure optimal integration of testing and device placement within facilities	Intervention fidelity varied across sites and was observed to be low in some settings	(n) PoC device utilization increased from 24% to 51% in Malawi facilities	N/A	N/A
Ganesh et al. (2020) Malawi. [30]	2 large HIV clinics	Descriptive analysis	2813 tests	2017-2019	same day actions	Quantitative	GeneXpert		N/A	N/A	N/A	The “all in” cost was $33.71 for a valid PoC-test compared centralized VL test of $28.62	(n)PoC VL test was feasible in high volume urban clinics, enabling prompt and effective clinical management	N/A	N/A	N/A	N/A
Villa et al., (2020) Ghana. [22]	Komfo Anokye Teaching Hospital	Prospective pilot	333	Feb 2018-Mar 2018	8 weeks	Quantitative	GeneXpert		N/A	N/A	N/A	N/A	(n)PoC was technically feasible with some bottlenecks like power supply and limited number of samples that can be run; targeted screening for those with signs of non-adherence	N/A	N/A	N/A	N/A
Drain et al., (2020) South Africa. [18]	CAPRISA and Prince Cyril Zulu Clinic	Open-label RCT (STREAM)	390 randomized	Feb 2017-Aug 2017	12months	Quantitative	GeneXpert	mPIMA	The study reported that PoC testing and task shifting was acceptable as participants could be referred directly for differentiated care	N/A	N/A	N/A	N/A	N/A	N/A	Sustainability requires innovative models of HIV care without extra burden on providers	N/A
Kufa et al., (2020) South Africa. [32]	4 tertiary obstetric units	Prospective implementation study	8147 births; 2912 VL	Jun 2018-March2019	Until discharge	Qualitative	GeneXpert	mPIMA	Benefits were observed such as identifying suboptimal viral suppression and lowering HIV transmission risk during peri and postpartum periods	N/A	N/A	N/A	Implementation success varies across sites, but requires more resources for adequate coverage and timely results. Highly feasible multi-disease tool to scale up HIV testing	After hours and weekend sampling decreased the proportion of same day return of results	Overall coverage of PoC VL testing was 35.6% (range 20% to 60.1%),partly caused by testing around birth being new and multiple reminders were necessary towards nurses	Scale up was needed to address health system factors such as high analytical error rates, optimal patient flows, placement of instruments to achieve best coverage and shorten turnaround time	N/A
Msimango et al. (2020) South Africa. [33]	Durban clinics	Qualitative sub study (STREAM)	55 clients +8 nurses	Mar-Aug 2018	Not applicable	Qualitative	GeneXpert		High satisfaction of (n)PoC test as it minimized the frequency of clinic visits, saving time, travel costs and days off work. For HCW, it was beneficial as they could immediately counsel	N/A	(n) PoC was appropriate, as same day results were available at the point of care and also supported task shifting and improved clinic efficiency	N/A	(n)PoC VL monitoring disrupted patient flow in a busy public clinic, with some unable to wait for results. HCWs raised a concern about staffing and quality assurance	N/A	N/A	N/A	N/A
Vasconcellos et al.(2020) Brazil. [21]	Federal University hospital lab	Validation study	149 specimens	2019	Not applicable	Quantitative		mPIMA	N/A	N/A	N/A	N/A	In a limited resource setting, it was a challenge to obtain 50 μL of plasma but in general, it was considered feasible	N/A	N/A	N/A	N/A
Girdwood et al. (2020) South Africa. [26]	NHLS facilities	Cost effectiveness modelling	Modelled > 5million VL samples/year	2018	Not applicable	Geospatial cost outcome	GeneXpert	mPIMA	Targeted testing was considered less acceptable to health care policy makers who seek to ensure equity in smaller clinics or improve access with long TAT and high specimen rejection rates	N/A	N/A	low PoC test costs enabled ART initiation and better health outcomes; reduced retention gains would raise costs but leave the annual VL budget unchanged	N/A	N/A	N/A	Low sustainability of (n)PoC test in clinics with challenges of staff availability and unreliable power supply	N/A
Simeon et al. (2019) South Africa. [20]	CAPRISA and Prince Cyril Zulu Clinic	Micro-costing (STREAM RCT)	390 randomized	2017-2018	12 months	Mixed method	GeneXpert	mPIMA	N/A	N/A	N/A	Implementing PoC tests for ART monitoring costs about $45 more per patient than centralized labs over 5 years, with smaller facilities facing higher costs. Mult disease testing could lower expenses	The authors conclude that PoC testing for HIV care and treatment can be feasibly implemented within clinics in South Africa	N/A	N/A	N/A	N/A
Girdwood et al. (2019) Zambia. [25]	Decentralized clinics	Geospatial modelling study	Model-based	2016-2019	Not applicable	Quantitative	GeneXpert		N/A	N/A	N/A	Combining on site PoC and hub facilities minimizes costs, while cost sharing, transport optimization and strategic PoC placement further reduce expenses	N/A	N/A	N/A	N/A	N/A
Nicholas et al. (2019) Malawi. [35]	Chiradzulu District Hospital and 4 ART clinics	Retrospective cohort	21400 eligible	Aug 2013-Jun 2017	Median 6.8 months	Quantitative	SAMBA I VL		Adherence support and safeguarding of effective regimens add to the appeal of PoC testing. Further, treatment switching was much higher than in centralised settings	N/A	N/A	N/A	Variable managerial commitment and clinician resistance to shifting from CD4 to VL testing affected performance; regular training, mentoring and VL focal points are recommended	There were difficulties in implementing the adapted three test failure algorithm, or the non-capture of VL tests in the electronic database	VL testing coverage was 85%. In some clinics for those adults on ART > 24 months, it was higher	N/A	N/A
De Necker et al.(2019) Kenya. [24]	Modelling GeneXpert vs centralized testing	Markov cost-effectiveness model	Model based	5 years	Model based	Quantitative	GeneXpert		N/A	N/A	N/A	Using the existing GeneXpert platform for VL testing reduced HIV transmission, OIs and costs in non-pregnant individuals and children but increased transmission, OIs and costs in pregnant women	N/A	N/A	N/A	N/A	N/A
Ndlovu et al. (2018) Zimbabwe. [34]	3 rural facilities	Prospective feasibility	3160 samples	Nov 2015-Aug 2016	Not applicable	Quantitative	GeneXpert		Users found the GeneXpert system easy to use, providing VL results in 90 minutes and reported high satisfaction without added workload	High uptake of (n)PoC for its immediate provision of results	N/A	N/A	Implementing (n)PoC GeneXpert for mult- disease testing is feasible and improves VL access but faces challenges like strict sample requirements, infrastructural needs, limited staff and potential device overload	High levels of proficiency among all trained staff were observed	N/A	N/A	GeneXpert requires minimal training and biosafety measures, but stronger TB-HIV program collaboration is needed to optimize resources, services and costs
Estill et al. (2013) South Africa. [29]	Gugulethu and Khayelitsha ART programmes	Mathematical modelling	9888 ART patients	17.8 months median	Until switch failure	Quantitative	GeneXpert		N/A	N/A	N/A	(n)PoC VL cost effectiveness was limited but improved with higher detection limit, fewer new infections and reduced ART failure through targeted counselling	N/A	N/A	N/A	N/A	N/A

nPoC describes near point of care. DH describes district hospital, HC describes health centre, PoC VL describes point of care viral load, RH describes referral hospital, ART describes Antiretroviral Therapy, NHLS describes the National Health Laboratory Service, RCT describes a randomised control trial, TAT describes the Turn around time, OIs describes Opportunistic infections, N/A describes not applicable, N describes Sample size

### Study characteristics

Twenty papers were included in this systematic review, all of which were conducted in low and middle-income countries [17–36]. Among the studies, sixteen were quantitative studies, [18,19,21–31,34–36] two were qualitative studies, [32,33] and two were mixed methods studies [17,20]. Other study characteristics, including country, setting, study design, sample size, duration and follow-up period are shown in Table 1.

### Overall results

We assessed the risk of bias using the Newcastle-Ottawa Scale [15]. Twelve studies had a low risk of bias (scored 7–9) [17–22,24–27,31,36]. These studies utilized well-defined representative populations, measured outcomes using reliable tools, had longer follow-up periods, and showed low rates of loss to follow-up. Seven studies had an unclear risk of bias (scored 4–6) [23,28–30,32,33,35]. These studies lacked randomization or appropriate control group selection and did not sufficiently adjust for confounding factors. Additionally, five studies had short follow-up periods, while two studies reported higher rates of loss to follow-up. Only one study had a high risk of bias and scored 3; this was the prospective feasibility study conducted in Zimbabwe, which highlighted potential bottlenecks [34] (S5 Table). The study’s selection bias reduces its generalizability, with a high risk of bias arising from the lack of randomization and the choice of control groups. Furthermore, the short follow-up period combined with the high loss to follow-up rate further reduces the reliability of the findings

Each study described at least one of the eight IROs defined by Proctor et al [13].

### Acceptability

Eight studies described the acceptability of (n)PoC VL monitoring [18,26,28,31–35].

Studies in Malawi, Uganda and South Africa (SA) on (n) PoC VL monitoring, using SAMBA I in Malawi and Uganda and GeneXpert in South Africa, reported high satisfaction due to reduced turnaround times from blood draw to results and at the same time minimizing clinic visits for high-risk patients [31,33]. Further, in the South Africa(SA) study, POC testing and task shifting were acceptable as they enabled direct referral to differentiated care [18] In addition, another SA study reported that acceptability was also linked to benefits such as identifying suboptimal viral suppression and lowering HIV transmission risk during peri and postpartum periods [32]. In a study conducted in Zimbabwe, HCWs felt comfortable carrying out (n)POC VL monitoring independently, and laboratory staff found it easy to use, with viral load results accessible within 90 minutes [34]. In the Malawian study, patients reported (n)POC VL monitoring as effective for adherence support and safeguarding of effective regimens, adding to the appeal of POC testing. Further treatment switching was much higher than in centralised settings [35].

However, targeted testing was perceived as less acceptable to some healthcare policymakers who seek to ensure equity by extending access to smaller clinics or by improving access to patients at low-volume clinics with long turnaround times (TAT) and high specimen rejection rates [26]. Additionally, the Ugandan study found that participants disliked waiting at the clinic to receive their results, reflecting the stigma associated with VL testing and limited knowledge about the testing process [28]. In conclusion, PoC VL monitoring was highly acceptable with limited clinic access, long waits, and stigma as possible obstacles.

### Adoption

One study described the adoption of (n)PoC VL monitoring [34].

Medical professionals and patients observed a high uptake of (n)PoC VL monitoring in a study conducted in Zimbabwe for its immediate provision of results and counselling to patients, including multi-disease testing, which increases system efficiency [34]. Thus, it can be concluded that efficiency is the key factor that determines the uptake of (n)PoC VL monitoring in most clinics,

### Appropriateness

Appropriateness was touched upon in one study [33].

We hereby focus on whether POC addresses the problem of centralized testing, which leads to long turnaround times and the risk of losing samples or results. In an SA study (n), PoC VL monitoring was appropriate, as same day results were available at the point of care and also supported task shifting and improved clinic efficiency [33]. Overall, (n)PoC VL monitoring has been shown to be appropriate in limited resource settings.

### Costs

Costs were described in nine studies [17,19,20,24–26,29–31].

An observational study of (n)PoC VL monitoring conducted in Kenya revealed high costs in low-testing-volume clinics, with per-test costs of USD 54.93 compared to USD 24.25 in high-volume clinics, and the centralised testing costs were USD 25.65 per test [17]. Also, a study conducted in Malawi and Uganda reported that implementing (n)POC VL monitoring in numerous remote health facilities is perceived as unrealistic and too costly for Ministries of Health [31]. A Malawian study reported that the “all-in” cost was $33.71 for a valid (n)POC VL test compared with an international benchmark for a centralised VL test [30]. In addition, a study conducted in SA found that PoC tests for VL monitoring costs about $45 more per patient annually than centralized testing over five years; costs are higher in smaller clinics but may drop significantly with multi-disease GeneXpert testing [20]. Conversely, a study conducted in SA showed that the (n)POC VL monitoring intervention is cost-effective in moderately sized clinics [19]. Other studies conducted in SA showed low costs of POC test that led to faster ART initiation and improved overall health outcomes [26,29]. It was further noted that reductions in retention/suppression improvements would result in a higher cost per additional person suppressed, but the annual viral load budget estimated would remain the same [26]. Also, a study conducted in Zambia reported that an optimised combination of onsite POC placement and facilities acting as POC hubs had the lowest costs of POC tests [25]. From a Kenyan study, it was noted that using the existing GeneXpert for TB testing to conduct VL testing led to fewer HIV transmissions in non-pregnant individuals, fewer opportunistic infections among children and lower costs of VL monitoring. In pregnant women, it led to more transmission and more opportunistic infections and costs since VL was tested much more frequently [24]. The findings showed that (n)PoC HIV VL monitoring has higher costs in low-volume clinics and is more cost-effective in moderate-sized clinics, especially when implemented with the multi-disease testing tool.

### Feasibility

Feasibility was described in fourteen studies [17,19–23,28,30–36].

The Uganda study authors conclude that implementation of (n)PoC VL monitoring is feasible based on the overall results of the study, and they didn’t see utility disruptions or supply shortfalls [28]. Also, a study conducted in Haiti, the authors conclude, (n)PoC VL monitoring was efficiently integrated in care [36]. In Malawi and Uganda, a study reported that (n)PoC VL monitoring was more practical and effective due to faster turnaround time of results, although it faced logistical and staffing challenges; further, it was concluded that SAMBA1 VL can feasibly be set up in peripheral health facilities and district hospitals [31]. Another study conducted in Malawi and Zimbabwe reported that multiple-disease testing tools were highly feasible to scale up HIV testing [23]. In the Ghana study, same-day (n)PoC VL monitoring was technically feasible, with some bottlenecks like power supply and a limited number of samples that can be run; therefore, targeted screening for those with signs of non-adherence [22]. In the Kenya study, the authors conclude that (n)PoC VL monitoring can be feasibly implemented, but there is low feasibility in some settings that share the same instrument, causing delays in VL results [17]. In SA studies, implementation success varies across sites. One study showed POC VL and EID testing around delivery is feasible but requires more resources for adequate coverage and timely results [32]. Another study reported that in a Public clinic, (n)POC VL monitoring disrupted patient flow, with some unable to wait for the results. Healthcare workers also raised concerns about staffing and quality assurance [33]. A study conducted in Brazil showed that in a limited resource setting, it was a challenge to obtain 50 µL of plasma, but in general, it was considered feasible [21]. Malawians’ study reported that uneven managerial commitment likely affected site performance. Clinicians struggled to shift from CD4 to VL monitoring. Ongoing training, mentoring and VL focal points were recommended [35].

Near PoC testing was feasible in a study conducted in rural Zimbabwe, in district and subdistrict healthcare settings, but required infrastructure upgrades, proper sample handling and adequate staffing to avoid overburdening workers [34]. Point of care was shown to be feasible in South African clinics, though costs were higher in smaller clinics and could be reduced with the integration of multi-disease testing [20]. In addition, PoC testing was feasible and cost-effective in moderately sized SA clinics, especially when combined with client-centred care and differentiated service delivery [19]. Further, (n)PoC targeted VL testing was feasible in high-volume urban clinics in Malawi, enabling prompt and effective clinical management [30]. In conclusion, (n)PoC VL monitoring is feasible with the multi-disease testing; however, there was a concern of delays due to sharing the same instrument. Ensuring sufficient resources, infrastructure and staffing are needed for effective implementation.

### Fidelity

Fidelity was described in seven studies [23,27,28,31,32,34,35].

In the study conducted in Zimbabwe it was reported a high level of proficiency among all trained staff was observed [34]. In the study conducted in Uganda, it revealed that HIV viral load tests were never carried out because participants did not stay to have blood drawn, and others did not wait for their results [28]. A multi-country study, Cameroon, Congo, Senegal, Kenya and Tanzania, reported a number of system gaps within programmes that were identified for improvements, including results documentation at the facilities, results communication to patients and follow-up [27]. In a study in Malawi and Uganda, it was found that only 2% of test results for patients seen at one site were reviewed with the patient on the same day [31]. Also, in Malawi and Zimbabwe, studies showed that fidelity varied across sites and was observed to be low in some settings [23]. In the SA study, it was reported that after-hours and weekend sampling decreased the proportion of same-day return of results. [32] In the Malawian study it was reported that there was no capture of VL tests in the electronic database. [35] In conclusion, low fidelity was noted due to patients not waiting for results, poor documentation of results and variations in implementation across sites.

### Penetration

Four studies reported penetration [23,31,32,35].

In the study conducted in Malawi and Uganda, low penetration of (n)PoC VL monitoring was reported due to insufficient manpower and high workload, but task shifting to community HCWs may overcome these challenges [31]. Utilization of the (n)POC device increased from 24% to 51% in Malawi and from41% to 55% in Zimbabwe [23] Overall, the coverage of POC VL testing in a SA study was 35.6% [32]. While in a study conducted in Malawi, VL testing coverage was 85% in some clinics for those on ART [35]. The findings demonstrate that PoC VL monitoring has high coverage across studies, although there was low penetration in certain settings due to limited manpower and high workloads. However, a suitable allocation of responsibilities may help improve utilisation.

### Sustainability

Sustainability was described in four studies [18,26,31,32].

In the South African study supported sustainability through innovative models of HIV care only if they do not increase the existing burden on HIV care providers, laboratories and health system [18]. Further, scaling up of POC was observed in another SA study, which reported the need to address health system factors such as high analytical error rates, optimal patient flows, placement of instruments to achieve best coverage and shorten turnaround time [32].

On the other hand, the qualitative studies in SA, SAMBA studies in Malawi and Uganda, observed low sustainability of (n)PoC VL monitoring in clinics with challenges of staff availability and unreliable power supply [26,31]. In summary, in order to ensure sustainable (n)PoC VL monitoring, challenges such as poor documentation, staff shortages and power supply issues need to be addressed carefully.

## Discussion

This review has accumulated valuable insights into how PoC VL monitoring was implemented in various LMICs settings and how specific implementation bottlenecks may limit proper implementation. Out of the twenty studies, twelve studies specifically used GeneXpert for (n)PoC VL monitoring, five studies employed both GeneXpert and mPIMA, two studies used SAMBA 1 VL and only one study focused on mPIMA PoC VL monitoring.

The findings demonstrated high acceptability and feasibility of PoC VL monitoring due to rapid turnaround times, same-day results, and improved antiretroviral therapy (ART) adherence monitoring. However, implementation outcomes, such as fidelity (the intervention being implemented as intended), penetration (the practice being integrated within the service setting, and sustainability (the newly implemented intervention being maintained), were frequently constrained by systemic challenges. Key barriers included insufficiently trained staff, knowledge gaps in interpreting results, workflow disruptions in high-volume clinics, power supply interruptions, equipment breakdowns, and poor documentation of results.

Contextual patterns showed that urban and high-volume clinics reported higher feasibility and acceptability due to better infrastructure, staff availability and patient flows. In contrast, rural and low-volume clinics reported greater implementation challenges, including higher costs and logistics barriers caused by limited resources and unreliable infrastructure.

Platforms performance varied across settings. GeneXpert was the most widely used and praised for its efficiency and ease of use. However, it faced some challenges, including running out of cartridges, machine setup complexity, and dependency on stable electricity. The mPIMA was a better fit for smaller clinics, but only if the staff were trained correctly, while SAMBA demonstrated potential for use in all sorts of settings, but required time and resources to establish. The implementation of PoC VL monitoring faced several bottlenecks that hindered its full adoption. One of the most frequent challenges was the inability of less trained staff to interpret results correctly, especially when laboratory personnel were unavailable. Similar findings were reported by Tun et al., where HCWs lack enough training to implement (n)PoC VL monitoring, causing delays in clinical decision making [7]. This was also noted in a study by Wang et al., where HCWs lacked training to implement (n)PoC VL monitoring tests effectively [23]. Moreover, one study showed that 8.3% of PoC VL tests were unsuccessful due to operational issues such as improper device handling and patient-related factors, indicating challenges beyond technical functionality [28]. Additional implementation barriers included poor quality control procedures, weak supply chains and equipment breakdowns [8]. Test failures were often attributed to sample-related problems, cartridge stockouts, and power supply interruptions [7,31]. Furthermore, there were delays in clinic flow in large public health facilities [8,33]. These logistical and infrastructural barriers threaten the consistency and reliability of PoC VL results.

Cost emerged as a barrier, particularly in low-volume clinics. However, in moderate to high-volume settings, PoC VL testing was found to be more cost-effective, particularly when integrated with multi-disease systems like GeneXpert, which reduced the result turnaround time [8,21] Similarly, a study by Ritchie et al. reported that although SAMBA minimises transport costs in rural settings, for sustainability, it requires a reliable supply of consumables, as well as ongoing training, which increases implementation costs [10]. However, adequate infrastructure support, including reliable electricity, is required to maintain cost efficiency [7].

Furthermore, insufficient manpower and high workloads were recognised as obstacles to the implementation of PoC VL monitoring. This is in accordance with a study Engel et al. who reported that nurses perceived (n)PoC implementation as an extra workload, leading to patients’ delays in waiting for the results [37].

In terms of sustainability, the review identified persistent issues, including a lack of power, staff turnover, poor documentation, and inconsistent supply of consumables. It was noted that long-term success depends on integration with national laboratory policies and ongoing government support [7,31]. In settings where PoC was aligned with national health strategies and integrated into routine services, implementation outcomes were more favourable and sustainable over time [38].

Addressing the implementation bottlenecks requires integrated strategies, structural task shifting models with ongoing training [9,33] and mentorship to address knowledge gaps and improve fidelity [39]. Implementing multi-disease testing platforms can help reduce costs [40] while prioritising device placement in high-yield sites with equitable rural access [17,25] and aligning PoC implementation with the national HIV policy framework to strengthen sustainability [33]. The findings provide actionable evidence to policymakers and implementers when scaling up PoC VL testing effectively.

This review has several limitations. The author considered only peer-reviewed published articles, did not include unpublished reviews and the period for the included studies was restricted. Studies published between 2013–2024 and limited databases were covered. English language search terms were used, which may have led to the omission of relevant research published in other languages. The lack of data from geographic regions outside low- and middle-income countries raises concerns about bias and the generalizability of the findings. In assessing the risk of bias using the Newcastle-Ottawa Scale, one feasibility study from Zimbabwe was found to have a high risk of bias. Although the study provided useful information on PoC VL test, its design limitations and high participant drop-out rate make it less reliable; therefore, we included it in the review, but interpreted the results cautiously.

The strength of this review is that we used the Proctor framework, which gives a comprehensive overview of implementation research outcomes. Gaps could suggest further research that can provide better insights into using PoC or (n)PoC VL monitoring in healthcare settings, human resources, continuous staff training and sustainable supply chain of PoC or (n)PoC facilities.

## Conclusion

Implementing PoC or (n)PoC testing for HIV VL monitoring was acceptable, feasible and can reach a broad population. However, high costs, limited fidelity, and lack of penetration and sustainability may hinder the use of the (n)PoC test in improving patient care and health outcomes. Our findings indicate different implementation bottlenecks, such as non-laboratory HCWs not knowing how to interpret results and clinic delays due to high patient volumes. Additional challenges include increased costs due to low attendance, complex multi-disease testing and test accuracy concerns. Insufficient manpower and high workloads also affect implementation. Point of care knowledge and training are needed to address problems related to implementation outcomes. This enables researchers, public health communities and policymakers to collaborate effectively in developing and testing evidence-based interventions.

### Recommendations

Point of Care VL monitoring provides rapid results followed by adherence counselling compared to (n)PoC VL monitoring. Rapid results provided by PoC VL monitoring improved its acceptability, leading to early detection of treatment failure, minimized clinic visits and improved patient outcomes. To address the implementation challenges of (n)PoC VL monitoring, prioritisation of clear diagnostic pathways and proper documentation are needed, as identified in this review. Further research should address gaps in healthcare settings, human resources, staff training and the sustainable supply chain for facilities that use (n)PoC tests. Additionally, future research should explore the impact of PoC VL monitoring implementation in different settings, particularly urban versus rural contexts, as data on this distinction remain limited.

## Supporting information

S1 Table Search Strategies. (PDF)

S2 Table PRISMA 2020 Checklists. (PDF)

S3 Table Studies identified in the literature search. (PDF)

S4 Table Reasons for exclusion. (PDF)

S5 Table Newcastle Ottawa Scale. (PDF)
