# Supplementary material for: Implementation of point of care HIV viral load monitoring for people living with HIV in low- and middle-income countries: A systematic review on implementation research outcomes
Source: PLoS One. 2026 Feb 4;21(2):e0313802. doi: 10.1371/journal.pone.0313802 (PMC12872026; doi:10.1371/journal.pone.0313802)
Supplement: S1 Table — (PDF) [file pone.0313802.s001.pdf]

S1 Table Search Strategy

Keywords 3/06/2024

1. Point of care test
2. HIV
3. Viral load
4. Acceptability
5. Feasibility
6. Appropriateness
7. Cost
8. Adoption
9. Fidelity
10. Treatment monitoring

PubMed search strategy (55hits)

1.	"Point of care testing" [mesh] OR "Testing, Point-of-Care*"[tiab] OR "Point-Of-Care Diagnostic Testing*" [tiab] OR "Point Of Care Diagnostic Testing*"[tiab] OR "Point-Of-Care Diagnostic Tests"[tiab] OR "Point Of Care Diagnostic Tests*"[tiab] OR "Point-Of-Care Diagnostic Test*"[tiab] OR "Point Of Care Tests*"[tiab] OR "Point-Of-Care Test*"[tiab] OR "Point Of Care Test*"[tiab] OR "Test, Point-Of-Care*"[tiab] OR "Tests, Point-Of-Care*"[tiab] OR "Point of Care Testing"[tiab] "Point-Of-Care Diagnostics*"[tiab] OR "Point Of Care Diagnostics*"[tiab] OR "Point-Of-Care Diagnostic*"[tiab] OR "Bedside Testing*"[tiab] OR "Testing, Bedside*" [tiab] OR POC [tiab]
2.	"HIV "[mesh] OR "HIV infections"[mesh] OR "Human Immunodeficiency Virus"[tiab] OR "Immunodeficiency Virus, Human*"[tiab] OR "Immunodeficiency Viruses, Human*"[tiab] OR "Virus, Human Immunodeficiency*"[tiab] OR "Viruses, Human Immunodeficiency*"[tiab] OR "Human Immunodeficiency Viruses"[tiab] OR "Human T Cell Lymphotropic Virus Type III*"[tiab] OR "Human T-Cell Lymphotropic Virus Type III*"[tiab] OR "Human T-Cell Leukemia Virus Type III*"[tiab] OR "Human T Cell Leukemia Virus Type III*"[tiab] OR "LAV-HTLV-III"[tiab] OR "Virus, Lymphadenopathy Associated*"[tiab] OR "Viruses, Lymphadenopathy Associated*"[tiab] OR "Human T Lymphotropic Virus Type III*"[tiab] OR "Human T-Lymphotropic Virus Type III*"[tiab] OR "Virus, AIDS*"[tiab] OR "Viruses, AIDS*"[tiab] OR "Acquired Immune Deficiency Syndrome Virus*"[tiab] OR "Acquired Immunodeficiency Syndrome Virus"[tiab] OR "HTLV-III"[tiab] OR "HIV Infection"[tiab] OR "HIV coinfection"[tiab] OR HIV[tiab]
3.	"Viral load" [mesh] OR "Load, Viral" [tiab] OR "Viral Burden*" [tiab] OR "Burden, Viral*" [tiab] OR "Virus Titer*" [tiab] OR "Titer, Virus*" [tiab]
4.	"Patient Acceptance of Health Care" [mesh] OR "Health Care Utilization"[tiab] OR "Utilization, Health Care"[tiab] OR "Patient Acceptance of Healthcare"[tiab] OR "Health Care Seeking Behavior"[tiab] OR "Acceptability of Health Care"[tiab] OR "Health Care Acceptability"[tiab] OR "Acceptability of Healthcare"[tiab] OR Patient Acceptance of Health Care[tiab] OR Acceptability[tiab]

5.	"Feasibility studies" [mesh] OR "Feasibility Study" [tiab] OR "Studies, Feasibility" [tiab] OR "Study, Feasibility" [tiab] OR feasibility[tiab]
6.	"Continuity of Patient Care"[mesh] OR "program evaluation"[mesh] OR "Care Continuity, Patient"[tiab] OR "Patient Care Continuity"[tiab] OR "Continuum of Care"[tiab] OR "Care Continuum"[tiab] OR "Continuity of Care"[tiab] OR "Care Continuity"[tiab] OR "Evaluation, Program"[tiab] OR "Evaluations, Program"[tiab] OR "Program Evaluations"[tiab] OR "Program Sustainabilit*"[tiab] OR "Sustainability Program"[tiab] OR "Program Effectiveness"[tiab] OR "Effectiveness, Program"[tiab] OR "Program Appropriateness"[tiab] OR "Appropriateness, Program"[tiab] OR "Penetration"[tiab]
7.	"Cost-Benefit Analysis" [mesh] OR "Analyses, Cost-Benefit"[tiab] OR "Analysis, Cost-Benefit"[tiab] OR "Cost-Benefit Analyses" [tiab] OR "Cost Benefit Analysis"[tiab] OR "Analyses, Cost Benefit"[tiab] OR "Analysis, Cost Benefit" [tiab] OR "Cost Benefit Analyses" [tiab] OR "Cost Effectiveness"[tiab] OR "Effectiveness, Cost"[tiab] OR "Cost-Benefit Data" [tiab] OR "Cost Benefit Data"[tiab] OR "Data, Cost-Benefit"[tiab] OR "Cost-Utility Analysis"[tiab] OR "Analyses, Cost-Utility" [tiab] OR "Analysis, Cost-Utility"[tiab] OR "Cost Utility Analysis"[tiab] OR "Cost-Utility Analyses"[tiab] OR "Economic Evaluation"[tiab] OR "Economic Evaluations"[tiab] OR "Evaluation, Economic"[tiab] OR "Evaluations, Economic"[tiab] OR "Marginal Analysis"[tiab] OR "Analyses, Marginal" [tiab] OR "Analysis, Marginal" [tiab] OR "Marginal Analyses" [tiab] OR "Cost Benefit"[tiab] OR "Costs and Benefits"[tiab] OR "Benefits and Costs"[tiab] OR "Cost-Effectiveness Analysis"[tiab] OR "Analysis, Cost-Effectiveness"[tiab] OR "Cost Effectiveness Analysis"[tiab] OR "Cost"[tiab]
8.	"Facilities and services Utilization" [mesh] OR "Facilities Utilization" [tiab] OR "Services Utilization" [tiab] OR "Services Utilizations" [tiab] OR "Adoption"
9.	fidelity
10.	treatment monitoring
11.	#1 AND #2 AND #3 AND (#4 OR #5 OR #6 OR #7 OR #8 OR #9 OR #10)

Scopus search strategy (242 hits)

1.	TITLE-ABS-KEY (point AND of AND care AND testing)
2.	TITLE-ABS-KEY (hiv)
3.	TITLE-ABS-KEY (viral AND load)
4.	TITLE-ABS-KEY (acceptability)
5.	TITLE-ABS-KEY (adoption)
6.	TITLE-ABS-KEY (appropriateness)
7.	TITLE-ABS-KEY (cost)
8.	TITLE-ABS-KEY (feasibility)
9.	TITLE-ABS-KEY (fidelity)

10.	TITLE-ABS-KEY (sustainability)
11.	TITLE-ABS-KEY (treatment AND monitoring)
12.	1 AND 2 AND 3<br><br>(TITLE-ABS-KEY (point AND of AND care AND testing)) AND (TITLE-ABS-KEY (hiv)) AND (TITLE-ABS-KEY (viral AND load))
13.	4 OR 5 OR 6 OR 7 OR 8 OR 9 OR 10 OR 11<br><br>(TITLE-ABS-KEY (acceptability)) OR (TITLE-ABS-KEY (adoption)) OR (TITLE-ABS-KEY (appropriateness)) OR (TITLE-ABS-KEY (cost)) OR (TITLE-ABS-KEY (feasibility)) OR (TITLE-ABS-KEY (fidelity)) OR (TITLE-ABS-KEY (sustainability)) OR (TITLE-ABS-KEY (treatment AND monitoring))
14.	12 AND 13<br><br>((TITLE-ABS-KEY (point AND of AND care AND testing)) AND (TITLE-ABS-KEY (hiv)) AND (TITLE-ABS-KEY (viral AND load))) AND ((TITLE-ABS-KEY (acceptability)) OR (TITLE-ABS-KEY (adoption)) OR (TITLE-ABS-KEY (appropriateness)) OR (TITLE-ABS-KEY (cost)) OR (TITLE-ABS-KEY (feasibility)) OR (TITLE-ABS-KEY (fidelity)) OR (TITLE-ABS-KEY (sustainability)) OR (TITLE-ABS-KEY (treatment AND monitoring)))

Cochrane search strategy (16 hit)

1.	("Point of care test"):ti,ab,kw
2.	("HIV"):ti,ab,kw
3.	("viral load"):ti,ab,kw
4.	#1 AND #2 AND #3
5.	("Acceptability"):ti,ab,kw
6.	("Adoption"):ti,ab,kw
7.	("Appropriateness"):ti,ab,kw
8.	("Cost"):ti,ab,kw
9.	("Feasibility"):ti,ab,kw
10.	("Fidelity"):ti,ab,kw
11.	("Sustainability"):ti,ab,kw
12.	("Treatment monitoring"):ti,ab,kw
13.	#5 OR #6 OR #7 OR #8 OR #9 OR #10 OR #11 OR #12
14.	#4 AND #13
