# Supplementary material for: Implementation of point of care HIV viral load monitoring for people living with HIV in low- and middle-income countries: A systematic review on implementation research outcomes
Source: PLoS One. 2026 Feb 4;21(2):e0313802. doi: 10.1371/journal.pone.0313802 (PMC12872026; doi:10.1371/journal.pone.0313802)
Supplement: S3 Table — (PDF) [file pone.0313802.s003.pdf]

S3 Table. Studies identified in the literature search

	First Author	Year of publication	Title
1.	Agutu	2019	Systematic review of the performance and clinical utility of point of care HIV-1 RNA testing for diagnosis and care
2.	Abuelezam	2019	Modelling the epidemiologic impact of achieving UNAIDS fast-track 90-90-90 and 95-95-95 targets in South Africa
3.	Bardon	2021	Simplifying TREAtment and Monitoring for HIV (STREAM HIV): protocol for a randomised controlled trial of point-of-care urine tenofovir and viral load testing to improve HIV outcomes
4.	Chaplin	2023	Timeliness of Point-of-Care Viral Load Results Improves Human Immunodeficiency Virus Monitoring in Nigeria
5.	Creese	2019	The development and use of the assessment of dementia awareness and person-centred care training tool in long-term care
6.	Dorward	2017	Protocol for a randomised controlled implementation trial of point-of-care viral load testing and task shifting: the Simplifying HIV TREAtment and Monitoring (STREAM) study
7.	Dorward	2021	Protocol for a randomised feasibility study of Point-Of-care HIV viral load testing to Enhance Re-suppression in South Africa: the POwER study
8.	Drain	2020	Point-of-care HIV viral load testing combined with task shifting to improve treatment outcomes (STREAM): findings from an open-label, non-inferiority, randomised controlled trial
9.	Hermans	2022	TENOFOVIR URINE POINT-OF-CARE TEST PREDICTS VIREMIA and DRUG RESISTANCE during ART

	First Author	Year of Publication	Title
10.	Kroidi	2023	BIRTH POINT-OF-CARE TEST & TREAT REDUCES EARLY MORTALITY AMONG HIV INFECTED INFANTS
11.	Mennecier	2021	Design and challenges of a large HIV prevention clinical study on mother-to-child transmission: ANRS 12397 PROMISE-EPI study in Zambia and Burkina Faso.
12.	Msimango	2020	Acceptability of point-of-care viral load testing to facilitate differentiated care: a qualitative assessment of people living with HIV and nurses in South Africa
13.	Nair	2019	Psychometric properties of the Child Oral-care Performance Assessment Scale
14.	Ochodo	2022	Point-of-care viral load tests to detect high HIV viral load in people living with HIV/AIDS attending health facilities
15.	Patel	2020	Optimizing viral load suppression in Kenyan children on antiretroviral therapy (Opt4Kids)
16.	Patel	2022	Point-of-care HIV viral load and targeted drug resistance mutation testing versus standard care for Kenyan children on antiretroviral therapy (Opt4Kids): an open-label, randomised controlled trial
17.	Alemnji	2020	Clinical/Laboratory Interface Interventions to Improve Impact of Viral Load and Early Infant Diagnosis Testing Scale-Up.
18.	Boeke	2021	Evaluation of near point-of-care viral load implementation in public health facilities across seven countries in sub-Saharan Africa.
19.	Ceffa	2016	Comparison of the Cepheid GeneXpert and Abbott M2000 HIV-1 real time molecular assays for monitoring HIV-1 viral load and detecting HIV-1 infection.

	First Author	Year of Publication	Title
20.	Chang	2023	Clinical Outcomes in a Randomized Controlled Trial Comparing Point-of-Care With Standard Human Immunodeficiency Virus (HIV) Viral Load Monitoring in Nigeria.
21.	Chibwesha	2016	Point-of-Care Virologic Testing to Improve Outcomes of HIV-Infected Children in Zambia: A Clinical Trial Protocol.
22.	Curtis	2018	A multiplexed RT-LAMP assay for detection of group M HIV-1 in plasma or whole blood.
23.	Drain	2017	Point-of-care diagnostics: extending the laboratory network to reach the last mile.
24.	Estill	2013	Cost-effectiveness of point-of-care viral load monitoring of antiretroviral therapy in resource-limited settings: mathematical modelling study.
25.	Fidler	2017	A pilot evaluation of whole blood finger-prick sampling for point-of-care HIV viral load measurement: the UNICORN study.
26.	Ganesh	2021	Near Point-of-Care HIV Viral Load: Targeted Testing at Large Facilities.
27.	Girdwood	2019	Optimizing viral load testing access for the last mile: Geospatial cost model for point of care instrument placement.
28.	Gueguen	2021	Implementation and operational feasibility of SAMBA I HIV-1 semi-quantitative viral load testing at the point-of-care in rural settings in Malawi and Uganda.
29.	Habiyambere	2016	Availability and Use of HIV Monitoring and Early Infant Diagnosis Technologies in WHO Member States in 2011-2013: Analysis of Annual Surveys at the Facility Level.

	First Author	Year of Publication	Title
30.	Ritchie	2014	SAMBA HIV semiquantitative test, a new point of care viral load monitoring assay for resource-limited settings
31.	Moirana	2022	Evaluation of HIV viral load turnaround time in Moshi, Tanzania
32.	Nakyanzi	2024	It soothes your heart. A Multimethod Study Exploring Acceptability of Point of Care Viral Load Testing among Ugandan Pregnant and Postpartum Women Living with HIV
33.	Dorward	2018	Point of care viral load testing and differentiated HIV care
34.	Stevens	2014	Feasibility of HIV point of care tests for resource-limited settings: Challenges and solutions
35.	Moyo	2016	Point of care Cepheid Xpert HIV-1 Viral Load Test in Rural African Communities Is Feasible and Reliable
36.	Qian	2022	After viral load testing, I get my results, so I get to know which path my life is taking me. qualitative insights on routine centralised and point-of-care viral load testing in western Kenya from the Opt4Kids and Opt4Mamas studies
37.	Avram	2019	Point of care HIV viral load in pregnant women without prenatal care: a cost-effectiveness analysis
38.	Drain	2019	Point of Care HIV Viral load Testing: an Essential Tool for a Sustainable Global HIV/AIDS Response
39.	Hull	2022	Isothermal Amplification with a Target-Mimicking Internal Control and Quantitative Lateral Flow Readout for Rapid HIV Viral Load Testing in Low-Resource Settings.

	First Author	Year of Publication	Title
40.	Hyle	2017	The value of point-of-care CD4+ and laboratory viral load in tailoring antiretroviral therapy monitoring strategies to resource limitations.
41.	Jani	2016	Evaluation of the Whole-Blood Alere Q NAT Point-of-Care RNA Assay for HIV-1 Viral Load Monitoring in a Primary Health Care Setting in Mozambique.
42.	Kabir	2020	Advances in HIV diagnosis and monitoring.
43.	Kamble	2023	A protocol for feasibility of plasma based GeneXpert platform and Dried Blood Spot (DBS) based Abbott platform for HIV-1 viral load testing among the people living with HIV attending ART centers in India.
44.	Khumalo	2020	The Cascade of Care From Routine Point-of-Care HIV Testing at Birth: Results From an 18-Months Pilot Program in Eswatini.
45.	Kufa	2020	Point-of-care HIV maternal viral load and early infant diagnosis testing around time of delivery at tertiary obstetric units in South Africa: a prospective study of coverage, results return and turn-around times.
46.	Lifson	2016	Advances in biosensing strategies for HIV-1 detection, diagnosis, and therapeutic monitoring.
47.	LO, Gora	2016	Prevalence of hepatitis B markers in Senegalese HIV-1-infected patients.
48.	Naing	2023	The feasibility and acceptability of integrating hepatitis C and HIV diagnostic testing on centralized molecular laboratory platforms in Myanmar.
49.	Ndlovu	2018	Multidisease testing for HIV and TB using the GeneXpert platform: A feasibility study in rural Zimbabwe.

	**First Author**	**Year of publication**	**Title**
50.	Ngo	2023	Sensitive and Quantitative Point-of-Care HIV Viral Load Quantification from Blood Using a Power-Free Plasma Separation and Portable Magnetofluidic Polymerase Chain Reaction Instrument
51.	Nicholas	2019	Point-of-care viral load monitoring: outcomes from a decentralized HIV programme in Malawi.
52.	Patel	2023	Impact of point-of-care HIV viral load and targeted drug resistance mutation testing on viral suppression among Kenyan pregnant and postpartum women: results from a prospective cohort study (Opt4Mamas).
53.	Pease	2018	On-chip quadruplex priming amplification for quantitative isothermal diagnostics.
54.	Peter	2017	Early antiretroviral therapy initiation: access and equity of viral load testing for HIV treatment monitoring.
55.	Peter	2017	Scaling up HIV viral load - lessons from the large-scale implementation of HIV early infant diagnosis and CD4 testing.
56.	Pham	2017	Feasibility of antiretroviral treatment monitoring in the era of decentralized HIV care: a systematic review.
57.	Philips	2024	Comparing a point-of-care urine tenofovir lateral flow assay to self-reported adherence and their associations with viral load suppression among adults on antiretroviral therapy.
58.	Reif	2022	Point-of-care viral load testing among adolescents and young adults living with HIV in Haiti: a randomized control trial.

	First Author	Year of Publication	Title
59.	Reif	2020	Point-of-care viral load testing among adolescents and youth living with HIV in Haiti: a protocol for a randomised trial to evaluate implementation and effect.
60.	Scott	2015	Laboratory evaluation of the Liat HIV Quant (IQuum) whole-blood and plasma HIV-1 viral load assays for point-of-care testing in South Africa.
61.	Shafiee	2015	Printed Flexible Plastic Microchip for Viral Load Measurement through Quantitative Detection of Viruses in Plasma and Saliva.
62.	Sharma	2021	Cost-effectiveness of point-of-care testing with task-shifting for HIV care in South Africa: a modelling study.
63.	Sher	2021	Development of a Point-of-Care Assay for HIV-1 Viral Load Using Higher Refractive Index Antibody-Coated Microbeads.
64.	Simeon	2019	Comparative cost analysis of point-of-care versus laboratory-based testing to initiate and monitor HIV treatment in South Africa.
65.	Steinmetzer	2010	HIV load testing with small samples of whole blood.
66.	Technau	2018	12-month outcomes of HIV-infected infants identified at birth at one maternity site in Johannesburg, South Africa: an observational cohort study.
67.	Vasconcellos	2020	Development and validation of a simple and rapid way to generate low volume of plasma to be used in point-of-care HIV virus load technologies.
68.	Engel	2017	Making HIV testing work at the point of care in South Africa: A qualitative study of diagnostic practices
69.	Violette	2022	Evaluation of SAMBA II: A Qualitative and Semiquantitative HIV Point-of-Care Nucleic Acid Test.
70.	Wang	2012	Efficient on-chip isolation of HIV subtypes.

	First Author	Year of Publication	Title
71.	Wang	2010	Advances in developing HIV-1 viral load assays for resource-limited settings.
72.	Wang	2024	Using queueing models as a decision support tool in allocating point-of-care HIV viral load testing machines in Kisumu County, Kenya.
73.	Yadav	2023	A SERS based clinical study on HIV-1 viral load quantification and determination of disease prognosis.
74.	Engel	2015	Compounding diagnostic delays: A qualitative study of point of care testing in South Africa.
75.	Ahn	2015	Effects of CD4 Monitoring Frequency on Clinical End Points in Clinically Stable HIV-Infected Patients with Viral Suppression
76.	Alemnji	2018	Improving Laboratory Efficiency in the Caribbean to Attain the World Health Organization HIV Treat All Recommendations
77.	Alemnji	2017	Improving laboratory efficiencies to scale-up HIV viral load testing
78.	Andrea	2019	Rapid point-of-care HCV RNA quantification in capillary whole blood for diagnosing chronic HCV infection, monitoring treatment and detecting reinfection
79.	Asio	2020	Population-based monitoring of HIV drug resistance early warning indicators in Uganda: A nationally representative survey following revised WHO recommendations
80.	Bachanas	2022	Protecting the gains: analysis of HIV treatment and service delivery programme data and interventions implemented in 19 African countries during COVID-19

	First Author	Year of Publication	Title
81.	Bacon	2023	Review of HIV Self Testing Technologies and Promising Approaches for the Next Generation
82.	Balakrishnan	2011	Low-cost assays for monitoring HIV infected individuals in resource-limited settings
83.	Barbara	2017	Optimizing Treatment Monitoring in Resource Limited Settings in the Era of Routine Viral Load Monitoring
84.	De Necker	2019	Economic and public health of decentralized HIV viral load testing: A modelling study in Kenya
85.	Barnabas	2020	Community-based antiretroviral therapy versus standard clinic-based services for HIV in South Africa and Uganda (DO ART): a randomised trial
86.	Bechini	2016	The role of the general practitioner in the screening and clinical management of chronic viral hepatitis in six EU countries
87.	Belec	2011	Challenges in implementing HIV laboratory monitoring in resource-constrained settings: How to do more with less
88.	Bender	2020	Integrated sample preparation for HIV molecular testing in a paper-based device
89.	Berry	2014	HIV viral RNA extraction in wax immiscible filtration assisted by surface tension (IFAST) devices
90.	Broucker	2021	The cost effectiveness of scaling up rapid point of care testing for early infant diagnosis of HIV in southern Zambia
91.	Botha	2018	Living donor liver transplant from an HIV-positive mother to her HIV-negative child: Opening up new therapeutic options

	First Author	Year of Publication	Title
92.	Bourlet	2023	Molecular testing for human immunodeficiency virus
93.	Boyce	2023	It takes more than a machine: A pilot feasibility study of point-of-care HIV-1 viral load testing at a lower-level health center in rural western Uganda
94.	Brazier	2021	Effects of National Adoption of Treat-All Guidelines on Pre-Antiretroviral Therapy (ART) CD4 Testing and Viral Load Monitoring After ART initiation: A Regression Discontinuity Analysis
95.	Bregenzer	2019	Rapid point-of-care HCV RNA quantification in capillary whole blood for diagnosing chronic HCV infection, monitoring treatment and detecting reinfection
96.	Brenner	2024	Projecting the Clinical and Economic Impacts of Changes to HIV Care Among Adolescents and Young Adults in the United States: Lessons From the COVID-19 Pandemic
97.	Buchholz	2002	German-Austrian recommendations for HIV-therapy in pregnancy–common declaration of The German AIDS-society (DAIG), The Austrian AIDS-society (OEAG) as well as The Robert-Koch Institute Berlin (RKI), The German Association of Physicians specialized in HIV Care (DAGNAE), The German Society of Pediatric and Youth Medicine (DGKJ), The German AIDS Pediatric Association (PAAD), The German Society of
98.	Bulterys	2021	Costs of point-of-care viral load testing for adults and children living with hiv in kenya
99.	Buriti	2013	Hearing loss in children with HIV/AIDS

	First Author	Year of Publication	Title
100.	Bystryak	2020	Preclinical assessment of a cartridge-based flow-through assay for determination of adult cd4 t-cell count
101.	Carmona	2017	HIV viral load scale-up: multiple interventions to meet the HIV treatment cascade
102.	Chamane	2022	The effect of a mobile-learning curriculum on improving compliance to quality management guidelines for HIV rapid testing services in rural primary healthcare clinics, KwaZulu-Natal, South Africa: a quasi-experimental study
103.	Chandane	2024	Revolutionizing HIV-1 Viral Load Monitoring in India: The Potential of Dried Blood Spot Analysis for Expanding Access and Improving Care
104.	Cogswell	2016	Viral-load point-of-care technologies to achieve an AIDS-free
105.	Craik	2016	Challenges with targeted viral load testing for medical inpatients at Queen Elizabeth Central Hospital in Blantyre, Malawi
106.	Damhorst	2013	Micro- and nanotechnology for HIV/AIDS diagnostics in resource-limited settings
107.	Deraney	2019	Vortex- and Centrifugation-Free Extraction of HIV-1 RNA
108.	Estill	2015	The cost-effectiveness of monitoring strategies for antiretroviral therapy of HIV infected patients in resource-limited settings: Software tool
109.	Evans	2014	CD4 criteria improves the sensitivity of a clinical algorithm developed to identify viral failure in HIV-positive patients on antiretroviral therapy

	First Author	Year of Publication	Title
110.	Facha	2021	Hiv/aids-related training coverage sponsored by centers for disease control and prevention and associated factors at health facilities providing antiretroviral therapy in southern Ethiopia
111.	Geretti	2009	HIV testing and monitoring
112.	Girdwood	2020	Cost-effectiveness of adoption strategies for point of care HIV viral load monitoring in South Africa
113.	Goel	2017	Performance of the SAMBA I and II HIV-1 Semi-Q Tests for viral load monitoring at the point-of-care
114.	Goga	2021	Eliminating HIV transmission through breast milk from women taking antiretroviral drugs
115.	Goldstein	2023	Person-centred, integrated non-communicable disease and HIV decentralized drug distribution in Eswatini and South Africa: outcomes and challenges
116.	Rasti	2017	Healthcare workers perceptions of point of care test in a low income country.A qualitative study in Southwestern Uganda
117.	Hellard	2020	Availability and Use of HIV Monitoring and Early Infant Diagnosis Technologies in WHO Member States in 2011–2013: Analysis of Annual Surveys at the Facility Level
118.	Ingole	2022	A randomized study of intensified antiretroviral treatment monitoring versus standard-of-care for prevention of drug resistance and antiretroviral treatment switch
119.	Ingole	2021	Comparison of CBNAAT and conventional real time RT PCR for HIV 1 viral load testing

	First Author	Year of Publication	Title
120.	Inzaule	2016	Affordable HIV drug-resistance testing for monitoring of antiretroviral therapy in sub-Saharan Africa
121.	Kahn	2011	CD4 cell count and viral load monitoring in patients undergoing antiretroviral therapy in Uganda: Cost effectiveness study
122.	Johannessen	2009	Dried blood spots can expand access to virological monitoring of HIV treatment in resource-limited settings
123.	Katzenstein	2003	Molecular biological assessment methods and understanding the course of the HIV infection
124.	Kanakasabapathy	2017	Rapid, label-free CD4 testing using a smartphone compatible device
125.	Khan	2023	Use of whole blood and dried blood spot for detection of HIV-1 nucleic acids using reverse transcription loop-mediated isothermal amplification
126.	Kimanga	2023	Impact of the COVID-19 pandemic on routine HIV care and antiretroviral treatment outcomes in Kenya: A nationally representative analysis
127.	Kosasih	2023	Evaluation of Rapid Molecular Viral Load Monitoring with Abbott TM m-PIMA HIV-1/2 VL
128.	Kroidi	2020	High turnaround times and low viral resuppression rates after reinforced adherence counselling following a confirmed virological failure diagnostic algorithm in HIV-infected patients on first-line antiretroviral therapy from Tanzania
129.	Lampejo	2013	HIV virology, testing and monitoring

	**First Author**	**Year of Publication**	**Title**
130.	Li,X	2023	Highly sensitive and rapid point-of-care testing for HIV-1 infection based on CRISPR-Cas13a system
131.	Liu	2023	Compact Point-of-Care Device for Self-Administered HIV Viral Load Tests from Whole Blood
132.	Lynen	2010	Monitoring for treatment failure in patients on first-line antiretroviral treatment in resource-constrained settings
133.	Maiers	2015	An investigation of fingerstick blood collection for pointof- care HIV-1 viral load monitoring in South Africa
134.	Majors	2017	Point-of-care diagnostics to improve maternal and neonatal health in low-resource settings
135.	Mangwiro	2014	Does provision of point-of-care CD4 technology and early knowledge of CD4 levels affect early initiation and retention on antiretroviral treatment in HIV-positive pregnant women in the context of Option B+ for PMTCT?
136.	Manoto	2018	Point of care diagnostics for HIV in resource limited settings: An overview
137.	Mauk	2017	Miniaturized devices for point of care molecular detection of HIV
138.	Mcinziba	2023	Perspectives of people living with HIV and health workers about a point-of-care adherence assay: a qualitative study on acceptability
139.	Meloni	2019	The role of point-of-care viral load monitoring in achieving the target of 90% suppression in HIV-infected patients in Nigeria: Study protocol for a randomized controlled trial
140.	Memmi	2016	Molecular Testing for Human Imunodeficiency Virus

	First Author	Year of Publication	Title
141.	Metz	2021	Data Architecture to Support Real-Time Data Analytics for the Population-Based HIV Impact Assessments
142.	Reif	2022	Point of care viral load testing among adolescents and young adults living with HIV in Haiti: a randomized control trial
143.	Mugabo	2017	Detection of drug-induced dyslipidaemia in HIV-positive patients treated with protease inhibitors in a South African district – A retrospective study
144.	Murtagh	2016	Molecular diagnostics for use in HIV/AIDS care and treatment in resource-limited settings
145.	Mutambanengwe	2022	Impact of Motivational Enhanced Adherence Counseling and Point-of-Care Viral Load Monitoring on Viral Load Outcome in Women on Life-Long ART: A Randomized Pilot Study
146.	Myers	2018	Comparing dedicated and designated models of integrating mental health into chronic disease care: Study protocol for a cluster randomized controlled trial
147.	Reid	2013	Tracking the progress of HIV: The impact of point-of-care tests on antiretroviral therapy
148.	Nash	2018	Performance of the xpert HIV-1 viral load assay: A systematic review and meta-analysis
149.	Ndembi	2020	Predictors of first-line antiretroviral therapy failure among adults and adolescents living with HIV/AIDS in a large prevention and treatment program in Nigeria
150.	Newman	2021	HIV-1 viral load testing in resource-limited settings: Challenges and solutions for specimen integrity
151.	Nguyen	2023	Performances of Dried Blood Spots and Point-of-Care Devices to Identify Virological Failure in HIV-Infected Patients: A Systematic Review and Meta-Analysis

	First Author	Year of Publication	Title
152.	Nichols	2019	Monitoring viral load for the last will it cost?
153.	Ochieng	2015	Implementation and operational research: Correlates of adherence and treatment failure among Kenyan patients on long-term highly active antiretroviral therapy
154.	Oodit	2022	Guidelines for Perioperative Care in Elective Abdominal and Pelvic Surgery at Primary and Secondary Hospitals in Low–Middle-Income Countries (LMIC's): Enhanced Recovery After Surgery (ERAS) Society Recommendation
155.	Pannus	2016	Sensitivity and specificity of dried blood spots for HIV-1 viral load quantification: A laboratory assessment of 3 commercial assays
156.	Wang	2021	Feasibility and impact of near point of care integrated tuberculosis/HIV testing in Malawi and Zimbabwe
157.	Peter	2017	Early antiretroviral therapy initiation: access and equity of viral load testing for HIV treatment monitoring
158.	Philips	2015	Sustainable HIV treatment in Africa through viral-load-informed differentiated care
159.	Preiser	2020	Pooled testing: A tool to increase efficiency of infant HIV diagnosis and virological monitoring
160.	Renju	2021	Influence of evolving HIV treatment guidance on CD4 counts and viral load monitoring: A mixed-methods study in three African countries
161.	Rhee	2015	HIV-1 drug resistance mutations: Potential applications for point-of-care Genotypic resistance testing

	First Author	Year of Publication	Title
162.	Roberts	2012	Challenges and opportunities for the implementation of virological testing in resource-limited settings
163.	Roberts	2016	Scale-up of Routine Viral Load Testing in Resource-Poor Settings: Current and Future Implementation Challenges
164.	Rowley	2014	Developments in CD4 and viral load monitoring in resource-limited settings
165.	Sangeda	2014	Pharmacy refill adherence outperforms self-reported methods in predicting HIV therapy outcome in resource-limited settings
166.	Shen	2011	Multiplexed quantification of nucleic acids with large dynamic range using multivolume digital RT-PCR on a rotational SlipChip tested with HIV and hepatitis C viral load
167.	Shrivastav	2019	Role of public-private partnerships in achieving UNAIDS HIV treatment targets
168.	Sikazwe	2019	Retention and viral suppression in a cohort of HIV patients on antiretroviral therapy in Zambia: Regionally representative estimates using a multistage-sampling-based approach
169.	Singano	2021	Leveraging routine viral load testing to integrate diabetes screening among patients on antiretroviral therapy in Malawi
170.	Spinelli	2024	Point-of-care urine tenofovir monitoring of adherence to drive interventions for HIV treatment and prevention
171.	Steegen	2010	A comparative analysis of HIV drug resistance interpretation based on short reverse transcriptase sequences versus full sequences
172.	Sukapirom	2022	Performance Evaluation of BD FACSPresto™ Near-Patient CD4 Counter for Monitoring Antiretroviral Therapy in HIV-Infected Individuals in Primary Healthcare Clinics in Thailand

	First Author	Year of Publication	Title
173.	Tassembedo	2024	Evaluation of the prevention of mother-to-child transmission of HIV programs at the second immunization visit in Burkina Faso and Zambia
174.	Teran	2021	Longitudinal Viral Load Monitoring Using Home-Collected Dried Blood Spot Specimens of MSM Living with HIV: Results from a Feasibility Pilot Study
175.	Trick	2022	Filtration-assisted magneto fluidic cartridge platform for HIV RNA detection from blood
176.	Tuthill	2024	Understanding mother-to-child transmission of HIV among mothers engaged in HIV care in Kenya: a case report
177.	Van der Elst	2022	"Facilitating HIV status adjustment: Qualitative insights from the Tambua Mapema proof-of concept study in Kenya"
178.	Venter	2017	Diagnosis and monitoring of HIV programmes to support treatment initiation and follow-up and improve programme quality
179.	Villa	2020	Determining virological suppression and resuppression by point-of-care viral load testing in a HIV care setting in sub-Saharan Africa
180.	Vogt	2015	Access to CD4 testing for rural HIV patients: Findings from a cohort study in Zimbabwe
181.	Wu	2012	Low-cost tools for diagnosing and monitoring HIV infection in low-resource settings [Outils à faible coût pour le diagnostic et le suivi de l'infection par le VIH dans des contextes de ressources limitées]
182.	Zaniewski	2021	Regression discontinuity analysis demonstrated varied effect of Treat-All on CD4 testing among Southern African countries
