# Supplementary material for: Implementation of point of care HIV viral load monitoring for people living with HIV in low- and middle-income countries: A systematic review on implementation research outcomes
Source: PLoS One. 2026 Feb 4;21(2):e0313802. doi: 10.1371/journal.pone.0313802 (PMC12872026; doi:10.1371/journal.pone.0313802)
Supplement: S4 Table — (PDF) [file pone.0313802.s004.pdf]

S4 Table Reasons for exclusion.

A total of 14 studies were excluded. Reasons for exclusion

First Author	Year of publication	Title	Reason for exclusion
Ritchie	2014	SAMBA HIV semiquantitative test, a new point of care viral load monitoring assay for resource limited settings	Wrong outcomes
Moirana	2022	Evaluation of HIV viral load turnaround time in Moshi, Tanzania	Wrong intervention
Nakyanzi	2024	It soothes your heart. A Multimethod Study Exploring Acceptability of Point of Care Viral Load Testing among Ugandan Pregnant and Postpartum Women Living with HIV	Wrong study design
Dorward	2018	Point of care viral load testing and differentiated HIV care	Wrong intervention
Stevens	2014	Feasibility of HIV point of care tests for resource-limited settings: Challenges and solutions	Wrong study design
Moyo	2016	Point of care Cepheid Xpert HIV-1 Viral Load Test in Rural African Communities Is Feasible and Reliable	Wrong outcomes
Qian	2022	After viral load testing, I get my results, so I get to know which path my life is taking me. qualitative insights on routine centralised and point-of-care viral load testing in western Kenya from the Opt4Kids and Opt4Mamas studies	Wrong outcome

Avram	2019	Point of care HIV viral load in pregnant women without prenatal care: a cost-effectiveness analysis	Wrong study design
Drain	2019	Point of Care HIV Viral load Testing: an Essential Tool for a Sustainable Global HIV/AIDS Response	Wrong intervention
Tembo	2022	Testing can Be Done Anywhere: A qualitative Assessment of Targeted Community Based Point of Care Early Infant Diagnosis of HIV in Lusaka, Zambia	Wrong intervention
Broucker	2021	The cost-effectiveness of scaling up rapid point of care testing for early infant diagnosis of HIV in Southern Zambia	Wrong intervention
Engel	2017	Making HIV testing work at the point of care in South Africa: a qualitative study of diagnostic practices	Wrong intervention
Engel	2015	Compounding diagnostic delays: a qualitative study of point of care testing in South Africa	Wrong intervention
Rasti	2017	Healthcare workers perceptions of point of care testing in a low income country-A qualitative study in Southwestern Uganda	Wrong intervention
