# Supplementary material for: Implementation of point of care HIV viral load monitoring for people living with HIV in low- and middle-income countries: A systematic review on implementation research outcomes
Source: PLoS One. 2026 Feb 4;21(2):e0313802. doi: 10.1371/journal.pone.0313802 (PMC12872026; doi:10.1371/journal.pone.0313802)
Supplement: S5 Table — (PDF) [file pone.0313802.s005.pdf]

S4 Table Newcastle Ottawa scale. The quality assessment of the non-randomized studies

Study Author (year)	Outcome	A. Selection (maximum of four stars)				Comparability (maximum of two stars)	C. Outcome (maximum of three stars)			Total (maximum of nine stars)
		1. Representativeness of the exposed cohort	2. Selection of the non-exposed cohort	3. Ascertainment of exposure	4. Demonstration that outcome of interest was not present at start of study	1. Comparability of cohort based on the design or analysis	1. Assessment of outcome	2. Was follow-up long enough for outcomes to occur	3. Adequacy of follow-up of cohorts	
Boyce (2023)	Feasibility	★	☆	★	★	☆	★	★	★	6
Tembo (2022)	Acceptability	★	☆	★	★	☆	★	★	★	6
Ganesh (2021)	Feasibility	★	☆	★	☆	★	★	★	★	6
Wang (2021)	Feasibility, fidelity	★	☆	★	☆	★	★	★	★	6
Broucker (2021)	Cost	★	☆	★	★	★	★	★	☆	6
Boeke (2021)	Appropriateness, Sustainability	★	★	★	★	★	★	★	★	8
Bulterys (2021)	Cost, feasibility	★	★	★	★	★	★	★	★	8
Gueguen (2021)	Adoption, Feasibility, penetration, sustainability	★	★	★	★	★	★	★	☆	7
Sharma (2021)	Acceptability, sustainability	★	★	★	★	★	★	★	★	8
Kufa (2020)	Feasibility, penetration	★	☆	★	★	★	★	★	☆	6
Villa (2020)	Acceptability	★	★	★	★	★	★	★	★	8
Vasconcellos (2020)	Feasibility	★	☆	★	★	★	★	★	★	7

S4 Table Newcastle Ottawa scale. The quality assessment of the non-randomized studies

Study Author (year)	Outcome	A. Selection (maximum of four stars)				Comparability (maximum of two stars)	C. Outcome (maximum of three stars)			Total (6maximum of nine stars)
		1. Representativeness of the exposed cohort	2. Selection of the non-exposed cohort	3. Ascertainment of exposure	4. Demonstration that outcome of interest was not present at start of study	1. Comparability of cohort based on the design or analysis	1. Assessment of outcome	2. Was follow-up long enough for outcomes to occur	3. Adequacy of follow-up of cohorts	
Msimango (2020)	Acceptability, Appropriateness	★	★	★	☆	★	★	☆	★	6
Girdwood (2020)	Cost, Sustainability	★	★	★	☆	★	★	★	★	7
Girdwood (2019)	Adoption, Appropriateness	★	★	★	☆	★	★	★	★	7
Nicholas (2019)	Acceptability	★	★	★	☆	★	★	☆	★	6
Simeon (2019)	Cost	★	★	★	★	★	★	★	★	8
Necker (2019)	Cost	★	★	★	★	★	★	★	★	8
Ndlovu (2018)	Adoption, Appropriateness, cost	☆	★	☆	☆	★	★	☆	☆	3
Engel (2015)	Acceptability, Adoption, cost, Feasibility, penetration	★	★	★	★	★	★	☆	★	7
Engel (2017)	Acceptability, Fidelity, sustainability	★	★	★	★	★	★	☆	★	7
Rasti (2017)	Acceptability, Adoption	★	★	★	☆	★	★	☆	★	6
Estill (2013)	Cost, Sustainability	☆	☆	★	☆	★	★	★	☆	4

S4 Table Newcastle Ottawa scale. The quality assessment of the non-randomized studies

Study Author (year)	Outcome	A. Selection (maximum of four stars)				Comparability (maximum of two stars)	C. Outcome (maximum of three stars)			Total (6maximum of nine stars)
		1. Representativeness of the exposed cohort	2. Selection of the non-exposed cohort	3. Ascertainment of exposure	4. Demonstration that outcome of interest was not present at start of study	1. Comparability of cohort based on the design or analysis	1. Assessment of outcome	2. Was follow-up long enough for outcomes to occur	3. Adequacy of follow-up of cohorts	
Reif (2022)	Acceptability	★	★	★	★	★★	★	★	★	9
Drain (2020)	Acceptability, Feasibility	★	★	★	★	★★	★	★	★	9
